# Inhibitory effect of thymol on pheromone-mediated attraction in two pest moth species

**DOI:** 10.1038/s41598-020-79550-1

**Published:** 2021-01-13

**Authors:** Sergio López, Aroa Domínguez, Ángel Guerrero, Carmen Quero

**Affiliations:** Department of Biological Chemistry, Institute of Advanced Chemistry of Catalonia (CSIC), Jordi Girona 18-26, 08034 Barcelona, Spain

**Keywords:** Entomology, Chemical ecology

## Abstract

Plant essential oils are considered as important bio-sources for the development of natural and environmentally safe pest control tools due to their multiple modes of action on insects. In this paper we have evaluated the activity of commercially available thyme oil and its constituents thymol, carvacrol, and *p*-cymene, as potential disruptants of the pheromone-mediated communication in the major pest moths *Spodoptera littoralis* Boisduval (Lepidoptera: Noctuidae), and *Grapholita molesta* (Busck) (Lepidoptera: Tortricidae). In electroantennographic assays, the antennal response of males to thyme oil, thymol, and carvacrol was altered at high doses (10^3^–10^4^ µg), shifting the signal waveform into a biphasic negative–positive potential that caused a decay in the response. In wind tunnel assays, pheromone-mediated attraction of males of both species was interrupted in presence of thyme oil. Further trials demonstrated that thymol alone reduced the number of *G. molesta* and *S. littoralis* males landing on the pheromone source. This effect did not differ from that of thyme oil, although the latter provoked a significant reduction on downwind behavior steps in *S. littoralis*. Overall, our findings provide a preliminary basis for delving into the effect of thyme oil, and especially of its major constituent thymol, as potential mating disruptants of both species.

## Introduction

One of the main challenges of IPM programs is to continuously seek and develop novel eco-friendly and biorational strategies to minimize the application of synthetic pesticides for their side effects. In this context, the use of plant essential oils (EOs) for the development of sustainable and environmentally safe control strategies has gained great attention during the last two decades^1^. Essential oils are complex mixtures of secondary volatile compounds from plant metabolism, mainly composed of terpenes and terpenoids, and, to a lesser extent, by aliphatic and aromatic constituents of low molecular weight (aldehydes, alcohols, phenols, methoxy derivatives, and methylenedioxy compounds)^2^. Their structural diversity has promoted a vast research on the bioactivity of EOs on insects, as they exert feeding^3,4^ and oviposition deterrence^5,6^, repellency^7^, and toxicity in eggs^8^, immature stages^9–11^, and adults^12^.

Within the broad spectrum of aromatic plant EOs studied, most of them belong to the families Myrtaceae, Lauraceae, Lamiaceae, and Asteraceae^13^. Among Lamiaceae, common thyme *Thymus vulgaris* has been proved effective against pest insects. Thyme is an aromatic evergreen shrub native to the Mediterranean region, which has been used in traditional medicine since ancient times^14^. Some of the thyme components are the isomers thymol (2-isopropyl-5-methylphenol) and carvacrol (5-isopropyl-2-methylphenol), both deriving from the precursor *p*-cymene^15^, another constituent of *T. vulgaris*. In terms of repellency, several authors have reported the effect of thyme on different insect orders, including Diptera^16–18^, Coleoptera^19^, Hemiptera^20,21^, and Thysanoptera^22^. In this regard, the two phenolic monoterpenes thymol and carvacrol have been suggested to be repellent for different insect species^18,23–25^. In mosquitoes (Diptera: Culicidae), for instance, the repellency evoked by thyme on *Culex pipiens pallens* Coquillet is attributable to both phenolic monoterpenes and α-terpinene^18^, while the repellent effect of three Lamiaceae species against *Aedes albopictus* Skuse is associated to carvacrol^25^.

Despite the considerable literature on the repellent effect of thyme oil or constituents, to the best of our knowledge no research has been focused on a possible effect of this EO on insect pheromone communication. It is well known that the male attraction to sex pheromones can be disrupted by synthetic pheromone analogs^26–29^, and naturally-occurring compounds, such as volatiles and EO components from non-host plants^30–35^. Based on this, we wonder whether thyme would interfere in the pheromone-mediated attraction of the major pest moths *Spodoptera littoralis* Boisduval (Lepidoptera: Noctuidae) and *Grapholita molesta* (Busck) (Lepidoptera: Tortricidae)*.* The Egyptian cotton leafworm, *S. littoralis*, is a serious pest of cotton and many other crops^36^. In turn, the oriental fruit moth *G. molesta* is a primary pest of stone and pome fruits worldwide^37^. Traditionally, pheromone-based communication has been one major target for the management of both species, including attract-and-kill, mass trapping and mating disruption^38,39^. In this context, mating disruption has become a widespread control approach during the last decades. Therefore, the development of new natural mating disruptants could open promising avenues for environmentally-friendly management of both species. In this paper, we have tested commercially available thyme oil, and three of its major components, i.e., thymol and carvacrol, for their proven repellent properties, and their precursor *p*-cymene. The effect induced by these chemicals on the male chemoreception system of both species was assessed by electroantennography (EAG), and the possible antagonism to the pheromone response of thyme oil, single compounds and binary/ternary mixtures was evaluated in a wind tunnel.

## Results

### Chemical composition

GC–MS analysis of thyme oil revealed the presence of 17 compounds. The major components were thymol (45%) and *p*-cymene (30%), followed by three minor compounds: γ-terpinene (5%), linalool (4%), and carvacrol (3%) (See Supplementary Fig. S1 online). These five compounds accounted for 87% of the total composition, while each of the remaining 12 minor chemicals was present in less than 3%.

### Electroantennographic response

**Figure 1:**
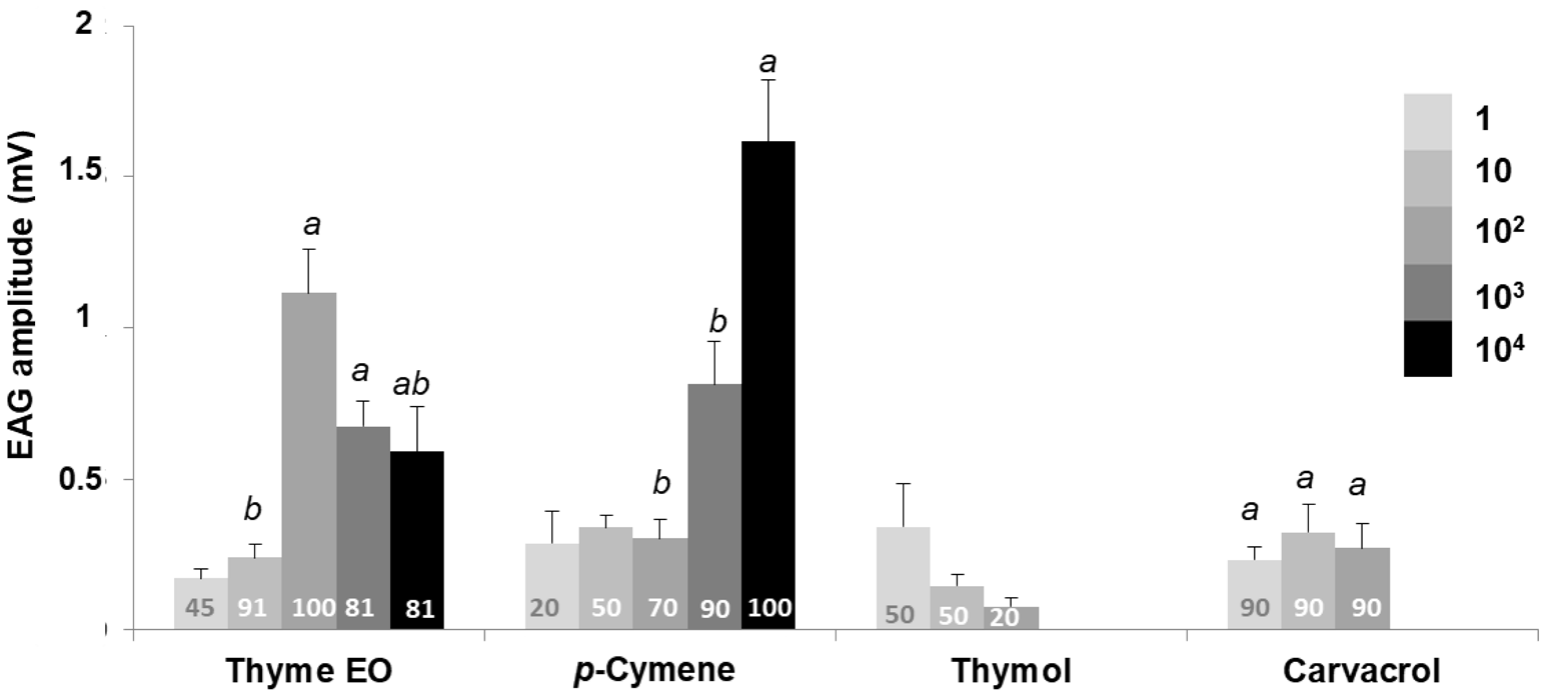
Mean electroantennographic response (mV + SEM) of *G. molesta* males (n = 10–11) to five different doses (1–10^4^ µg) of thyme oil, *p*-cymene, thymol and carvacrol. The number within each column indicates the percentage of antennae responding to the stimulus, that is, those which showed a net EAG response after subtraction of the solvent response. Columns lacking lower case letters indicate that less than 60% of tested antennae responded to the stimulus. Different letters within each treatment denote significant differences among doses (Kruskal–Wallis test followed by Mann–Whitney *U* test with Bonferroni correction).

**Figure 2:**
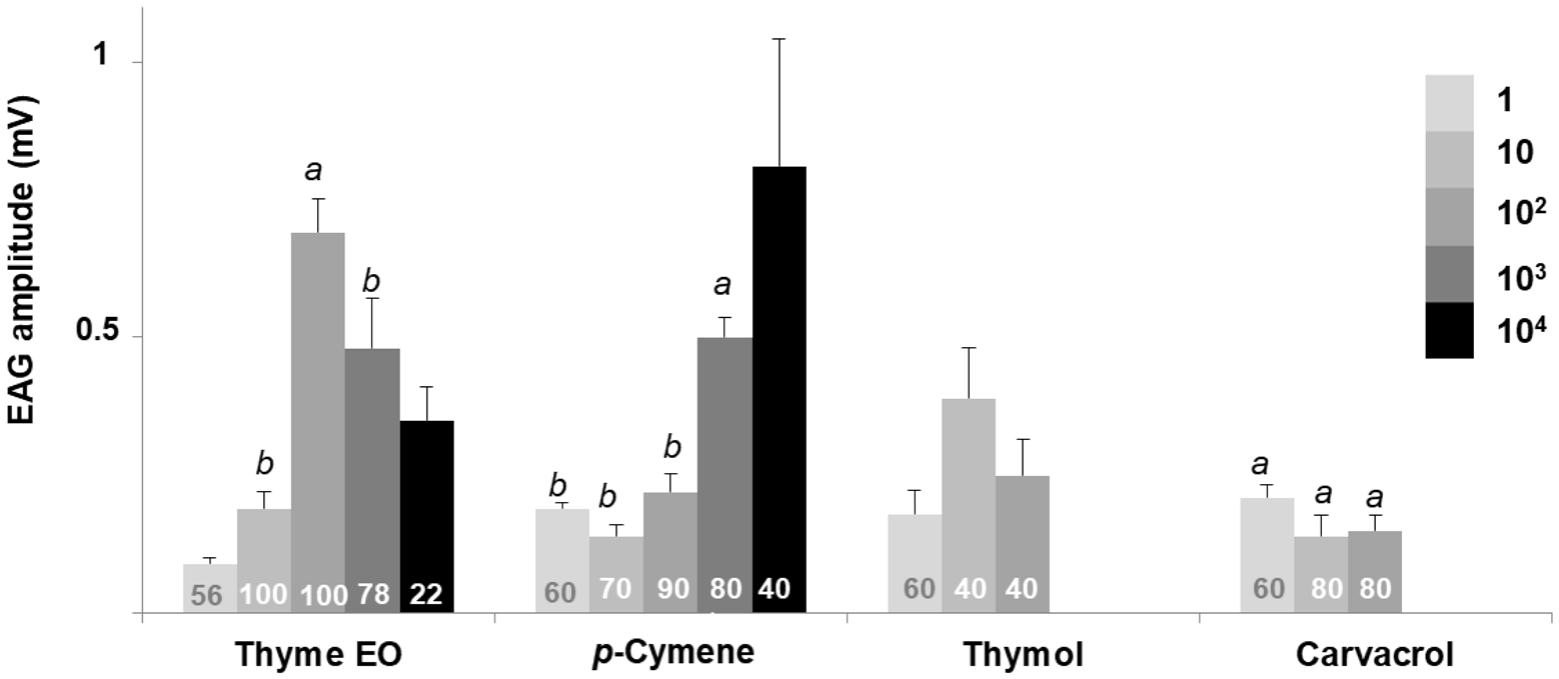
Mean electroantennographic response (mV + SEM) of *S. littoralis* males (n = 10–11) to five different doses (1–10^4^ µg) of thyme oil, *p*-cymene, thymol and carvacrol. The number within each column indicates the percentage of antennae responding to the stimulus, that is, those which showed a net EAG response after subtraction of the solvent response. Columns lacking lower case letters indicate that less than 60% of tested antennae responded to the stimulus. Different letters within each treatment denote significant differences among doses (Kruskal–Wallis test followed by Mann–Whitney *U* test with Bonferroni correction).

**Figure 3:**
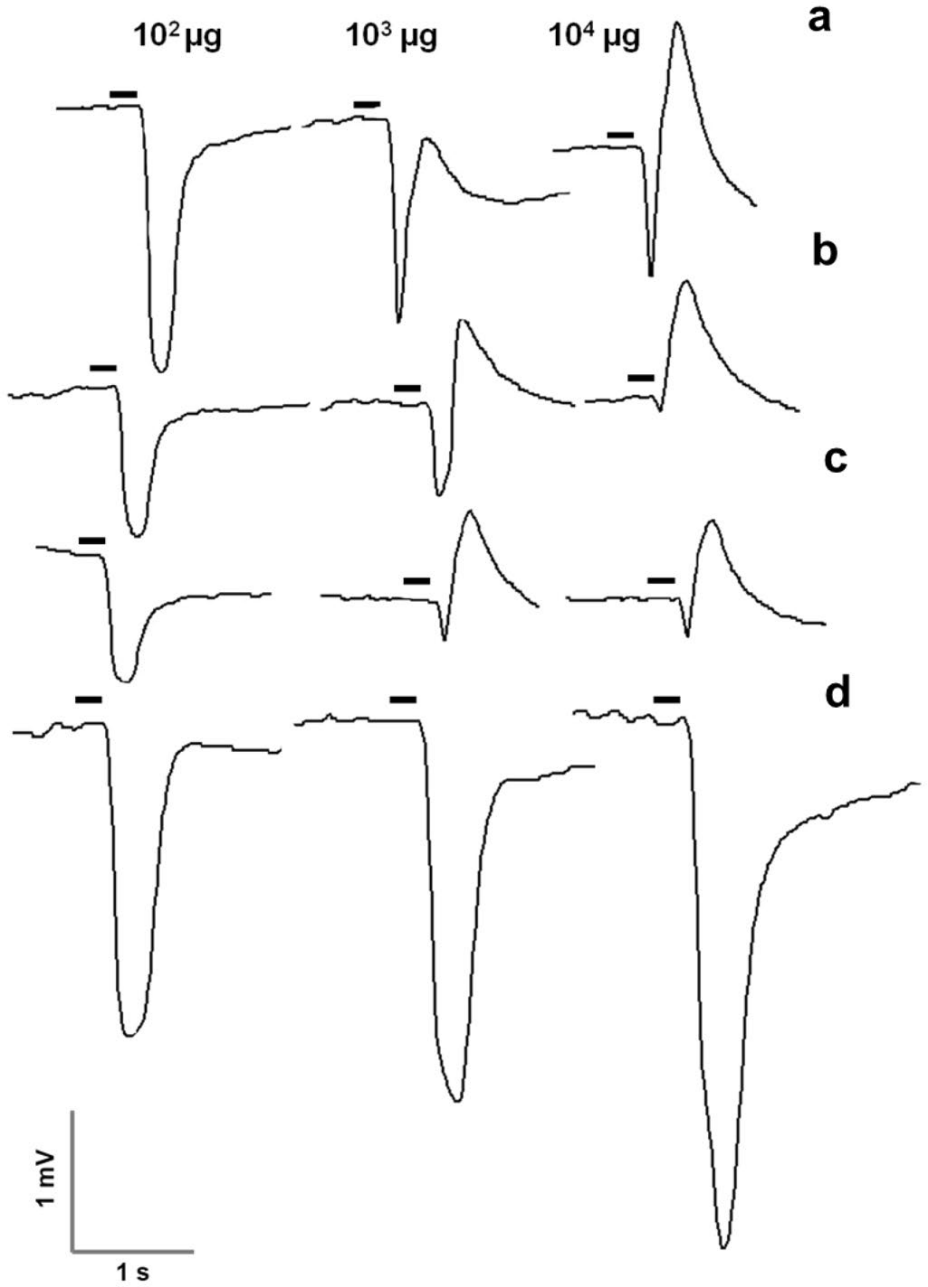
Representative EAG traces of *S. littoralis* males in response to 10^2^–10^4^ µg of thyme oil (**a**), carvacrol (**b**), thymol (**c**), and *p*-cymene (**d**). Horizontal bars over traces indicate duration of the puffed stimulus.

The EAG amplitudes in response to thyme oil and three of the constituents are displayed in Figs. 1 and 2. Significant differences in response were observed within thyme oil doses in both species, following a similar response pattern (*G. molesta*, χ^2^ = 17.726, df = 3, *p* = 0.001; *S. littoralis*, χ^2^ = 15.175, df = 2, *p* = 0.001). Males exhibited a dose-dependent response at low doses (1–10^2^ µg), and reached the maximum EAG amplitude at 10^2^ µg (*G. molesta,* 1.12 ± 0.15 mV; *S. littoralis,* 0.69 ± 0.06 mV), with less than 60% of the antennae of both species responding at 1 µg. However, a clear drop in the antennal response was observed at 10^3^ and 10^4^ µg. Indeed, a remarkable change in the shape of the response was observed at the highest dose in both species, with a small depolarization followed by a more pronounced positive polarization. Representative shape changes of *S. littoralis* males antennae to different treatments are shown in Fig. 3. Only two out of nine antennae responded at the highest dose *in S. littoralis*. In the case of *G. molesta*, no significant differences were detected among doses higher than 10 µg, and, even though the EAG response decayed at the highest dosages (10^3^–10^4^ µg), the percentage of antennae responding to 10^4^ µg was higher (81%) than in *S. littoralis*.

With regard to *p*-cymene, a dose-dependent effect was detected in both species (*G. molesta*, χ^2^ = 17.037, df = 2, *p* < 0.001; *S. littoralis*, χ^2^ = 19.058, df = 3, *p* < 0.001) (Figs. 1 and 2 respectively). *Spodoptera littoralis* antennae showed a lower response than *G. molesta*, although more antennae of the former insect (60–70% vs. 20–50%) responded at the lowest doses (1–10^2^ µg). This trend shifted at the highest dose (10^4^ µg) with only 40% of the antennae responding (0.81 ± 0.23 mV), in contrast to the 100% of *G. molesta* (1.62 ± 0.20 mV).

The EAG profiles of thymol and carvacrol differed from those obtained with *p*-cymene. Males of both species only responded to low doses (≤ 10^2^ µg), and the number of antennae sensitive to both stimuli was also relatively low, especially for thymol (less than 60%) almost all doses. Regarding carvacrol, no significant differences were detected between the active doses (1–10^2^ µg) in either species (*G. molesta*, χ^2^ = 1.058, df = 2, *p* = 0.589; *S. littoralis*, χ^2^ = 3.781, df = 2, *p* = 0.151). Here again, depolarization recordings of both phenolic compounds changed dramatically at 10^3^ and 10^4^ µg in both species (Fig. 3b,c shows representative traces of *S. littoralis*). In *G. molesta*, a similar change of the electroantennographic potential was noticed at the two highest doses of carvacrol and thymol, although the antennal recovery after the positive polarization did not return to the baseline, as similarly occurred with the thyme oil stimulus (see Supplementary Fig. S2 online).

### Wind tunnel

**Figure 4:**
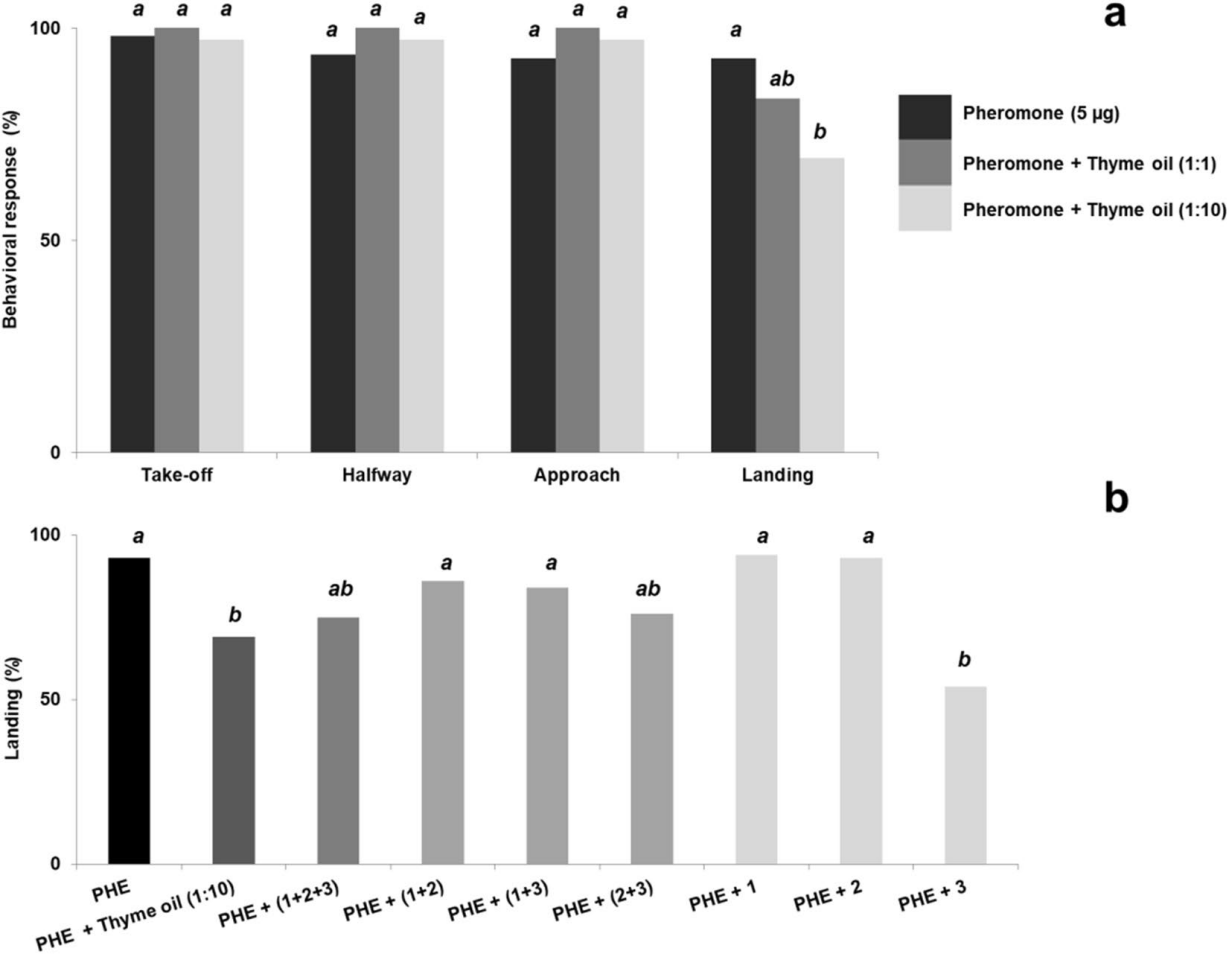
Sequential behavioral steps of *G. molesta* males in response to sex pheromone alone (PHE: 5 µg of *Z*8-12:OAc + *E*8-12:OAc + *Z*8-12:OH in 100:6:10 ratio) (n = 60), and co-released with thyme oil in 1:1 and 1:10 ratios (n = 30) (**a**); Landing percentage of males subjected to mixtures of PHE + thyme oil (1:10), PHE + *p*-cymene (15 µg) (**1**), PHE + carvacrol (1.5 µg) (**2**), PHE + thymol (22.5 µg) (**3**), and PHE + binary/ternary blends of thyme oil components (n = 30 for each test) (**b**). Bars with different letters are significantly different (Chi-square 2 × 2 test of independence with Yates’ correction, at α = 0.05).

Flight response of *G. molesta* males to the pheromone (5 µg of the mixture *Z*8-12:OAc, *E*8-12:OAc, and *Z*8-12:OH, in 100:6:10 ratio) in presence of thyme oil at 1:1 and 1:10 ratios was not affected for any of the behaviors except in landing, which significantly decreased from 93 to 69% at 1:10 ratio (χ^2^ = 7.024, df = 1, *p* = 0.008) (Fig. 4a, see also Supplementary Table S1 online for further details). In contrast, thyme oil at 1:1 ratio had no effect on the number of males contacting the source (χ^2^ = 1.250, df = 1, *p* = 0.264).

Subsequent trials were conducted by co-releasing the pheromone (5 µg) with single compounds in quantities present in 50 µg of thyme oil (thymol, 22.5 µg; carvacrol, 1.5 µg; *p*-cymene, 15 µg). Thymol significantly reduced to 54% the percentage of landings on the pheromone source (χ^2^ = 17.578, df = 1, *p* < 0.001), albeit this effect did not statistically differ from that elicited by the combination of thyme oil (50 µg) and pheromone (5 µg) (χ^2^ = 1.128, df = 1, *p* = 0.288) (Fig. 4b). No effect was detected for either carvacrol or *p*-cymene when singly released with the pheromone, with landing percentages of 94% and 93%, respectively.

The binary mixture of both phenolic compounds and the ternary blend performed similarly in combination with the pheromone. Both reduced the number of landing males to around 75%, close to significance with regard to the pheromone alone (χ^2^ = 3.741, df = 1, *p* = 0.053), although this effect did not differ from that of thymol (χ^2^ = 2.637, df = 1, *p* = 0.104). No effect was observed when binary blends containing *p*-cymene and carvacrol or thymol were tested (landing percentages of 86% and 84%, respectively) (Fig. 4b).

**Figure 5:**
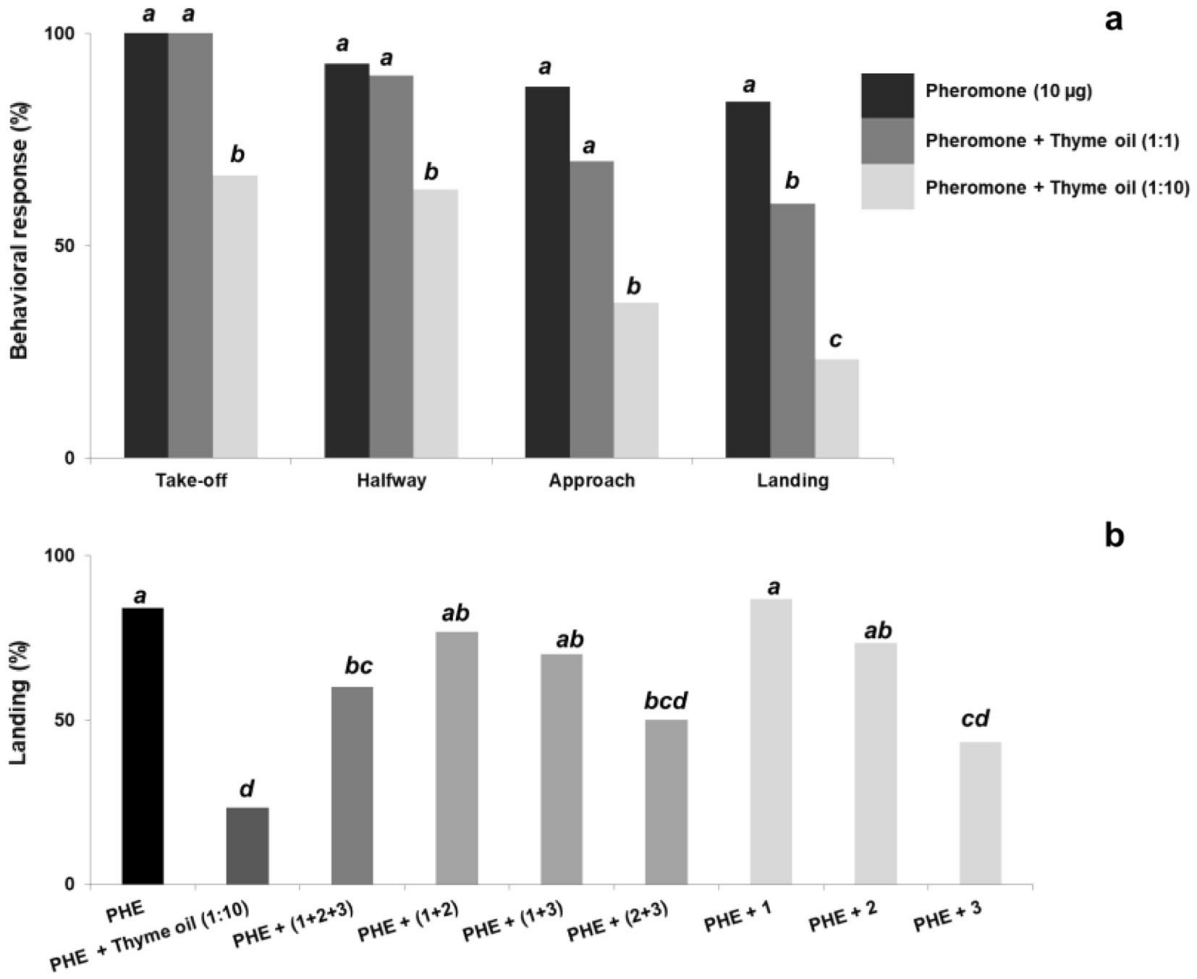
Sequential behavioral steps of *S. littoralis* males in response to the sex pheromone alone (PHE: *Z*9*E*11-14:OAc, 10 µg) (n = 56), or co-released with thyme oil at 1:1 and 1:10 ratios (n = 30) (**a**); Landing percentage of males subjected to mixtures of pheromone + thyme oil (1:10), PHE + *p*-cymene (30 µg) (**1**), PHE + carvacrol (3 µg) (**2**), PHE + thymol (45 µg) (**3**), and PHE + binary/ternary blends of thyme oil components (n = 30 for each test) (**b**). Bars with different letters are significantly different (Chi-square 2 × 2 test of independence with Yates’ correction, at α = 0.05).

Regarding *S. littoralis*, both pheromone (10 µg of *Z*9*E*11*-*14:OAc):thyme oil ratios inhibited the number of landing males, displaying significantly lower percentages than that elicited by 10 µg of the major component of the pheromone (84%) (Fig. 5a). Among treatments, the effect of 1:10 ratio was significantly higher than that exerted by 1:1 ratio (χ^2^ = 6.857, df = 1, *p* = 0.009). Thus, while the 1:1 ratio reduced the landing percentage to 60% (χ^2^ = 13.399, df = 1, *p* < 0.001), the 1:10 resulted in a four-fold decrease (23%) (χ^2^ = 32.641, df = 1, *p* < 0.001). Indeed, the 1:10 proportion significantly disrupted the remaining behavioral steps in response to the pheromone (take-off, χ^2^ = 18.003, df = 1, *p* < 0.001; halfway, χ^2^ = 9.864, df = 1, *p* = 0.002; approach, χ^2^ = 32.641, df = 1, *p* < 0.001) (Fig. 5a, Supplementary Table S2 online). As in *G. molesta*, additional tests were conducted by co-releasing the major pheromone component with the single constituents of thyme in the quantities present in 100 µg of the EO (thymol, 45 µg; carvacrol, 3 µg; *p*-cymene, 30 µg). Among the single components, thymol was the unique chemical displaying a disruptive effect, reducing the approach and landing scores to 67% (χ^2^ = 4.113, df = 1, *p* < 0.043) and 43% (χ^2^ = 13.399, df = 1, *p* < 0.001), respectively. Other downwind behavioral steps, i.e. take-off and halfway, were unaffected (see Fig. 5b, Supplementary Table S2 online). Even though thyme oil (1:10) induced a higher reduction in the number of landing males (23%), no significant differences were detected when compared to thymol (χ^2^ = 1.875, df = 1, *p* = 0.171). Approach to the source, in contrast, differed significantly between the whole EO mixture (37%) and thymol (χ^2^ = 4.271, df = 1, *p* = 0.039). The percentage of landing males was also decreased to 50% by the binary mixture of thymol and carvacrol (χ^2^ = 9.554, df = 1, *p* = 0.002), and the ternary blend (60%) (χ^2^ = 4.833, df = 1, *p* = 0.028), although neither treatment exerted the disruptive effect of thyme oil (50 µg) when mixed with the pheromone component. The presence of *p*-cymene in the tests either when singly co-released with the pheromone or in binary mixtures with thymol or carvacrol and the pheromone did not evoke any appreciable disruptant effect on any behavioral step. Indeed, it interrupted the activity of thymol alone, with 80% of males contacting the pheromone source when *p*-cymene was mixed with the former (χ^2^ = 1.420, df = 1, *p* = 0.233) (see Fig. 5b, Supplementary Table S2 online).

## Discussion

In most moth species, sexual communication is primarily ruled by female-released species-specific pheromones that provoke a stereotyped behavior on males. Even though insect olfactory systems are highly tuned for sensing mating chemical cues in fluctuating and complex odor landscapes, attraction to sex pheromone may be interrupted in presence of non-host volatiles. For example, it has been noticed in *S. littoralis* that the attractant effect of the pheromone in a wind tunnel is interrupted by volatiles of two non-host plants, namely *Picea abies* and *Adhatoda vasica*^40^. Likewise, other authors have reported that significantly fewer *G. molesta* males orient upwind towards a virgin calling female when the monoterpenoid citral is simultaneously released^31^. Similar activity of citral has also been reported for *Lobesia botrana* (Denis & Schiffermüller) (Lepidoptera: Tortricidae)^32^. In *Plutella xylostella* (L.) (Lepidoptera: Plutellidae), the terpenoids eucalyptol and *α*-terpineol suppress the attraction to the sex pheromone in wind tunnel and field trapping experiments^30^.

Here we provide evidence of the activity of thyme oil and its major component thymol as disruptors of the sex pheromone attractiveness on *G. molesta* and *S. littoralis* males. Thymol is abundantly found in EOs of Lamiaceae^41^, Verbenaceae^24^, and Apiaceae^23^, among other plant families. Toxic and repellent activity of thymol on arthropod pests of agricultural and medical-veterinary importance has been well documented^18,42^. For example, thymol is strongly repellent to the bean bug *Riptortus clavatus* (Thunberg) (Hemiptera: Alydidae)^23^, and *Sitophilus zeamais* (Motschulsky) (Coleoptera: Curculionidae)^24^. In *S. littoralis*, thymol is an acute toxic for larvae^43^, and also affects the behavior of adult females. In fact, a six-component blend containing thymol, carvacrol, benzaldehyde and three additional terpenes (eugenol, nerolidol and phytol) significantly deterred oviposition^44^. So far, this is the first time in which thymol is reported as an inhibitor of sex pheromone attraction of moths.

Herein, we demonstrate that in wind tunnel thymol inhibited landing on the pheromone source in *S. littoralis* and *G. molesta*. The number of the noctuid males approaching to the source was also diminished, while no effect was observed for this parameter in *G. molesta*. It should be pointed out that in both insects thymol was only evaluated at the amount contained in the corresponding pheromone:thyme oil ratio (1:10), and, therefore, additional research would be necessary to elucidate whether the effect of the chemical is dose-dependent. It is also remarkable that in *S. littoralis* thyme oil has a stronger effect than thymol in all behavioral steps considered. In addition, since binary and ternary blends of thymol with *p*-cymene and carvacrol did not improve the inhibitory response of thymol alone, the effect of thyme oil could be the result of a possible synergism of thymol with another minor constituent of the EO not considered in our bioassays. In this context, linalool, a monoterpene alcohol present in thyme oil (see Supplementary Fig. S1 online) that acts as a defense compound in damaged cotton plants, also interferes with the walking response of *S. littoralis* males to the major pheromone component^45^, and reduces the attraction of both genders towards host volatile blends^46^. In the same vein, (*E*)-4,8-dimethyl-1,3,7-nonatriene (DMNT), another herbivore-induced volatile, has been reported to exert disruption of *S. littoralis* behavior in response to pheromone and other attractant odors^46,47^. Physiological studies have revealed that these volatiles interfere with pheromone perception at peripheral level^45,47^. These results denote that volatiles from injured cotton plants act as ecologically significant signals interfering the sensory perception and, subsequently, modulating the behavior of both sexes. In addition and as mentioned before, volatiles from unsuitable or non-host plants may act as disruptive cues to avoid maladaptive choices and favor an efficient localization of conspecifics and hosts. In this sense, perception of the sex pheromone and host/non-host volatile plumes is a concomitant phenomenon that simultaneously converges into the olfactory system of the insect, with the volatiles affecting the sensitivity of pheromone olfactory receptor neurons (PHE-ORNs)^45,47–49^. Although plant volatile odorants are detected by other ORNs, it has been reported that some PHE-ORNs are activated by plant signals, possibly in a competing way^49,50^, and a concrete compound may differently act from one species to other. For instance, heptanal is known to activate PHE-ORNs in *Agrotis epsilon* (Hufnagel) (Lepidoptera: Noctuidae) males, while no activation is reported for *S. littoralis*^50^. Apart from direct activation, coding activity of the sex pheromone may be affected by the suppressive action of some volatiles. In the case of DMNT, the response of PHE-ORNs is reduced due to the suppression of the calcium activity^47^. As a consequence of these multimodal interactions of plant odorants in the pheromone detection process, antagonistic effects on the behavior are occasionally reported^45,47,51^. Considering our results, it is possible that thymol, a non-host volatile, may play a disruptive role on the pheromone detection at the peripheral level, leading to relevant changes in the sex pheromone-guided behavior. Nevertheless, the ecological significance of these interactions at PHE-ORNs level is still far from being fully understood.

The other constituents of thyme oil considered, i.e., *p*-cymene and carvacrol, did not exert any disruptant effect in either species at the doses present in the EO. Crude EO extracts have been proved to be more effective than individual components alone, owing to additive and/or synergistic interactions among constituents^43,52^. In our case, carvacrol did not induce any synergistic effect to the mixtures containing thymol. In contrast, *p*-cymene exerted an antagonistic effect when mixed with thymol, overriding the disruptive effect of the latter in both species. As previously stated, carvacrol by its own exerts repellency on insects^18,23,25^. Nonetheless, it must be taken into consideration that relatively low dosages (1.5–3 µg) were tested, and thus additional trials would be necessary to state consistent conclusions. Given the structural similarity between thymol and carvacrol, and considering that synergism depends not only on the suitable combination but also on the ratio of the components, a putative synergism between both phenolic compounds cannot be excluded.

Our EAG results showed that males of both species are capable of perceiving thyme oil and the three single compounds, although the response profile is notably different among compounds. Thus, the antennae of both species were more sensitive to *p*-cymene than to any other stimulus tested. While *p*-cymene induced electroantennographic activity in a dose-dependent manner, thyme oil, and particularly carvacrol and thymol, exhibited a notorious decrease in the antennal response at the highest doses. Indeed, the phenolic compounds (thymol and carvacrol) induced in both species moderate-to-low response at doses below 10^3^ µg, whereas no measureable EAG response was recorded at the highest stimuli. In a previous work, thymol evoked no EAG response in *S. littoralis* females at any of the doses tested (1–10^4^ µg), whereas the response to carvacrol decreased at the highest doses (10^2^–10^4^ µg)^44^. In our case, the drastic drop in the EAG response could be attributed to the change in the EAG waveform at the highest doses, characterized by a small negative potential followed by a large positive response. This effect was more acute when single compounds were puffed, since the effective dose of these stimuli represents a higher amount of compound than that contained in its equivalent thyme oil dose. Electroantennographic responses in insects are typically considered as single negative depolarizations, albeit the chemical structure of the odorants can alter the EAG shape^53^. This negative–positive response was firstly detected during the early development of the EAG technique^54^, while recording the effect of the exposure to chloroform on *Bombyx mori* (Lepidoptera: Bombycidae). Similarly, Light et al.^55^ recorded the same biphasic EAG shape on *Ceratitis capitata* (Wiedemann) (Diptera: Tephritidae) in response to short-chain acids. Some authors have suggested that the EAG response pattern can be correlated with behavioral activity of a particular compound. First evidence was reported for *Periplaneta americana* (L.) (Blattodea: Blattidae), in which those compounds generating positive polarizations were demonstrated to act as repellents^56^. Similar results were obtained by Ramachandran et al.^57^ in *Cnaphalocroeis medinalis* (Guenee) and *Marasmia patnalis* Bradley (Lepidoptera: Pyralidae). A stimulus of 10^2^ µg of either thymol or carvacrol evoked a negative–positive response in males and females of both species, and both compounds resulted to be an antifeedant for immature stages. Recently, Lee et al.^23^ obtained reversed EAG profiles from *R. clavatus* in response to 10^3^ µg of thymol and carvacrol, and both compounds were strongly repellent to females. These findings are partially in accordance with ours. Here, we suggest that the particular EAG shape at high doses may be correlated with an interference of the sexual communication in both species by reducing the attraction of males to the pheromone, specially in the case of thymol, which was proven to interrupt the behavior of males towards sex pheromone. Thus, we consider that thymol should be referred here as a disruptor of the pheromone attraction rather than a repellent. Nevertheless, as cited above, whether or not pheromone perception is affected by thymol at the peripheral level is unknown. In a similar vein, why a background of thyme oil constituents alters male innate orientation within the pheromone plume remains unanswered. In-depth electrophysiological recordings at the sensillum level combining pheromone, thymol, or other constituents, would help answer these questions. As outlined earlier, *S. littoralis* females did not respond to thymol in EAG assays, but single-sensillum recordings revealed the presence of two receptor neurons, one responding specifically to carvacrol, and another being sensitive to carvacrol, eugenol and thymol^44^.

The mechanisms underlying this shift in the EAG response profile are unclear. Thymol and carvacrol are known to affect the surface electrostatics and integrity of the cell membrane, where they can be incorporated and provoke membrane lesion and loss of permeability, leading to intracellular content leakage^58,59^. In our case, no deleterious sign on neurone membrane integrity was detected, since posterior stimuli to sex pheromone (1 µg) elicited a characteristic V-shape response in both species after the highest doses of thymol and carvacrol (Supplementary Fig. S3 online). Considering that thymol and carvacrol have been demonstrated to modulate second messengers (i.e. Ca^2+^, cAMP) level via tyramine receptors^60^, we suggest as a possible explanation that binding of thymol and carvacrol to receptors linked to the signal transduction pathway might provoke an alteration in second messenger cascade, which in turn would alter the EAG signal profile.

In conclusion, our study demonstrates that thyme oil disrupts the sex pheromone attraction in *S. littoralis* and *G. molesta*. This result represents a preliminary contribution for the development of novel practical approaches targeting pheromone communication of these two pest species. Further studies would be required to assess the potential of thyme oil or thymol as effective behavior manipulation tools under field conditions. In addition and to disclose the mechanism of the disruptive action of these chemicals, new electrophysiological experiments on specifically-tuned pheromone receptors would be necessary.

## Material and methods

### Insects

For *G. molesta*, a first batch of pupae was provided by the School of Agrifood and Forestry Science and Engineering—ETSEA (University of Lleida, Spain), to establish a new colony in our laboratory based on a previously described rearing methodology^61^.

In the case of *S. littoralis,* males were obtained from a long-term colony reared at the Institute of Advanced Chemistry of Catalonia—CSIC (Barcelona, Spain). Rearing methodology and maintenance have been described elsewhere^62^.

### Chemicals

The pheromone components of *G. molesta* (*Z*)-8-dodecenyl acetate (*Z*8-12:OAc) (≥ 97%) and (*E*)-8-dodecenyl acetate (*E*8-12:OAc) (≥ 96%) were prepared in our laboratory^63^, while (*Z*)-8-dodecen-1-ol (*Z*8-12:OH) (≥ 99%) was purchased from Pherobank (Wageningen, The Netherlands). The major sex pheromone component of *S. littoralis (Z,E)-*9,11-tetradecadienyl acetate (*Z*9*E*11*-*14:OAc, > 95% purity) was purchased from Bedoukian Research, Inc. (Danbury, CT, USA). Thyme oil and carvacrol (98%) were obtained from Merck/Sigma-Aldrich (Madrid, Spain). *p*-Cymene (> 97%) and thymol (> 98%) were acquired from Alfa Aesar (Karlsruhe, Germany).

### Chemical composition

Prior to behavioral assays, the chemical composition of thyme oil was analyzed using a GC (Thermo Finnigan Trace 2000 GC) coupled to a Trace MS quadrupole mass spectrometer (ThermoFisher Scientific, Madrid, Spain) equipped with a TR-5MS capillary column (30 m length, 0.25 mm i.d., film thickness 0.25 mm) (ThermoFisher Scientific). The percentage of *p*-cymene, carvacrol and thymol was estimated by peak area normalization. Additional compounds with an abundance of ≥ 3% were considered for identification by comparison of their mass spectra with those of reference compounds from NIST mass spectra library **(**NIST Chemistry WebBook, NIST Standard Reference Database Number 69), and synthetic standards.

### Electroantennography (EAG)

EAG assays were conducted following previous standardized procedures^64^. For sample preparation, each antenna was excised under a binocular stereo microscope, and the first 2–3 antennomeres of the tip were cut off. Then, the antenna was mounted on a forked microelectrode holder (Syntech, Kirchzarten, Germany), and both ends were fixed to the corresponding electrode with a drop of conductive gel (Spectra 360, Parker Lab. Inc., Hellendoorn, The Netherlands). The holder was connected to a MP-15 micromanipulator (Syntech), and placed 1.0 cm below the main branch of a glass tube (7 cm long × 5 mm diameter) that continuously blew humidified pure air (*ca*. 750 mL/min) over the antenna to keep it clean and prevent desiccation. The output signals were amplified (10×), filtered (DC to 1 kHz) with an IDAC-2 interface (Syntech), further amplified (10×), digitized on a PC and analyzed with the EAG Pro software Version 2.0 (Syntech).

Dose–response profiles for each treatment, *viz.* thyme oil, *p*-cymene, carvacrol, and thymol, were obtained by testing five doses (from 1 to 10^4^ µg) on antennae of 1–2 day old virgin males (*G. molesta* n = 10–11; *S. littoralis*, n = 9–10). To obtain the desired doses, decimal serial dilutions in analytical n-hexane were prepared from a stock solution (500 mg/mL) of each chemical, and 20 µL of the corresponding dilution were loaded on a filter paper strip (2.5 cm diameter, Whatman No.1 (Merck/Sigma-Aldrich). For delivering the stimulus, the filter paper was placed into a disposable glass Pasteur pipette. Stimuli were carried out by giving air puffs (ca. 200 mL/min and 200 ms duration) with the aid of a stimulus controller CS-01 (Syntech). Doses were tested from the lowest to the highest, and for each treatment two stimulations were applied on the antenna at 60 s intervals. Control puffs with a filter paper disc containing 20 µL of solvent that had been previously evaporated were intercalated between two consecutive stimuli to determine the mechanosensory response. The net EAG amplitudes (mV) were calculated by subtracting the mean response to the solvent before and after each stimulus from the mean response of the corresponding dose. Doses which did not exert a response on at least 60% of the antennae were excluded from statistical analysis. In the same way, when solvent subtraction from chemical response resulted in negative values, the corresponding recordings were also discarded.

### Wind-tunnel assays

Flight response of virgin males of first and second scotophase to sex pheromone alone or in combination with thyme oil and constituents was evaluated in a glass wind-tunnel (180 cm long × 55 cm wide × 50 cm high), as previously described^62^. An incoming filtered air speed of 30 cm/s was set. For *G. molesta*, appropriate illumination was obtained using a 58 W fluorescent white lamp located 16 cm above the tunnel, which provided *ca*. 100 lx inside the tunnel. For *S. littoralis* assays, the tunnel was illuminated by a similar 58 W red fluorescent light. All tests were conducted 2 h into the scotophase under a relative humidity of 30 ± 10%. The tunnel was thoroughly cleansed with 70% ethanol prior and after a set of assays.

The response to the sex pheromone alone in both species was daily assessed (*G. molesta,* n = 60; *S. littoralis,* n = 56), and for each trial including pheromone plus thyme oil or constituents in single/binary/ternary combinations, 30 males were tested. Prior to the beginning of each test, insects were individually isolated in Petri dishes (9 cm diameter), and acclimatized to the room conditions for 1 h. In all assays each male was used only once. Sex pheromone was co-released with thyme oil in 1:1 and 1:10 ratio, while the dosages for single constituents and binary/ternary mixtures were defined according to their percent composition in the EO. The pheromone and mixtures of the chemicals were loaded on Whatman filter paper discs (2.5 cm diameter) separated by 2 cm, approximately. For *G. molesta*, 5 µg of the sex pheromone blend of *Z*8-12:OAc, *E*8-12:OAc, and *Z*8-12:OH in 100:6:10 ratio^61^ was used. On the other hand, 10 µg of *Z*9*E*11*-*14:OAc were used as attractant for *S. littoralis*, since we reported earlier that the major component alone is capable of eliciting the full behavioral sequence in wind tunnel assays^62^. Filter papers were replaced every day. *Grapholita molesta* males were allowed to respond for 2 min^61^, and *S. littoralis* males for 5 min^62^. Four responding behaviors were scored: (1) take-off from the release platform at 110 cm downwind from the odor source, (2) upwind flight up to half-way of the tunnel, (3) approach to the source, and (4) landing. Landing was positively annotated when the insect remained more than 5 s in contact with the source.

### Statistical analysis

Differences in absolute EAG amplitudes within a treatment were analyzed by the non-parametric Kruskal–Wallis test, at a significance level of α = 0.05. When significant differences were detected, pairwise comparisons among doses were subjected to the Mann–Whitney *U* test. Bonferroni correction (α/number of comparisons) was applied to adjust probability values. Behavioral steps in the wind tunnel were analyzed by Chi-square 2 × 2 test of independence with Yates' correction. All tests were performed using SPSS Statistics 17.0.

## Supplementary Information

Supplementary Information.
