# Supplementary material for: Inhibitory effect of thymol on pheromone-mediated attraction in two pest moth species
Source: Sci Rep. 2021 Jan 13;11:1223. doi: 10.1038/s41598-020-79550-1 (PMC7807059; doi:10.1038/s41598-020-79550-1)
Supplement: Supplementary file 1 — Supplementary Information. [file 41598_2020_79550_MOESM1_ESM.pdf]

Supplementary Information (SI)

Inhibitory effect of thymol on pheromone-mediated attraction in two pest moth species

Sergio López†*, Aroa Domínguez†, Ángel Guerrero, Carmen Quero*

Department of Biological Chemistry, Institute of Advanced Chemistry of Catalonia (CSIC), Jordi Girona 18-26, 08034 - Barcelona, Spain.

†Both authors contributed equally to the work

*To whom correspondence should be addressed. E-mail: carme.quero@cid.csic.es, sergio.lopez@cid.csic.es

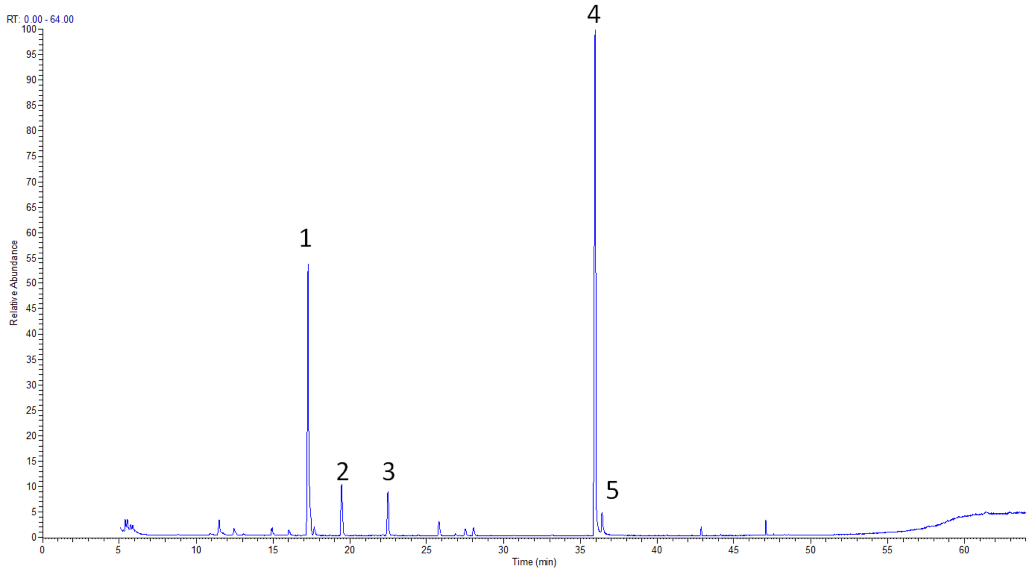

Figure S1 Chromatographic profile of commercial thyme oil. Only compounds with a percentage higher than 3% are shown: *p*-cymene (**1**), γ-terpinene (**2**), linalool (**3**), thymol (**4**), and carvacrol (**5**). Temperature program: 50 ºC for 1 min, 3 ºC/min to 150 ºC, then 5 ºC/min to 200 ºC, and finally raised at 10 ºC/min to 300 ºC, with a hold time of 10 min.

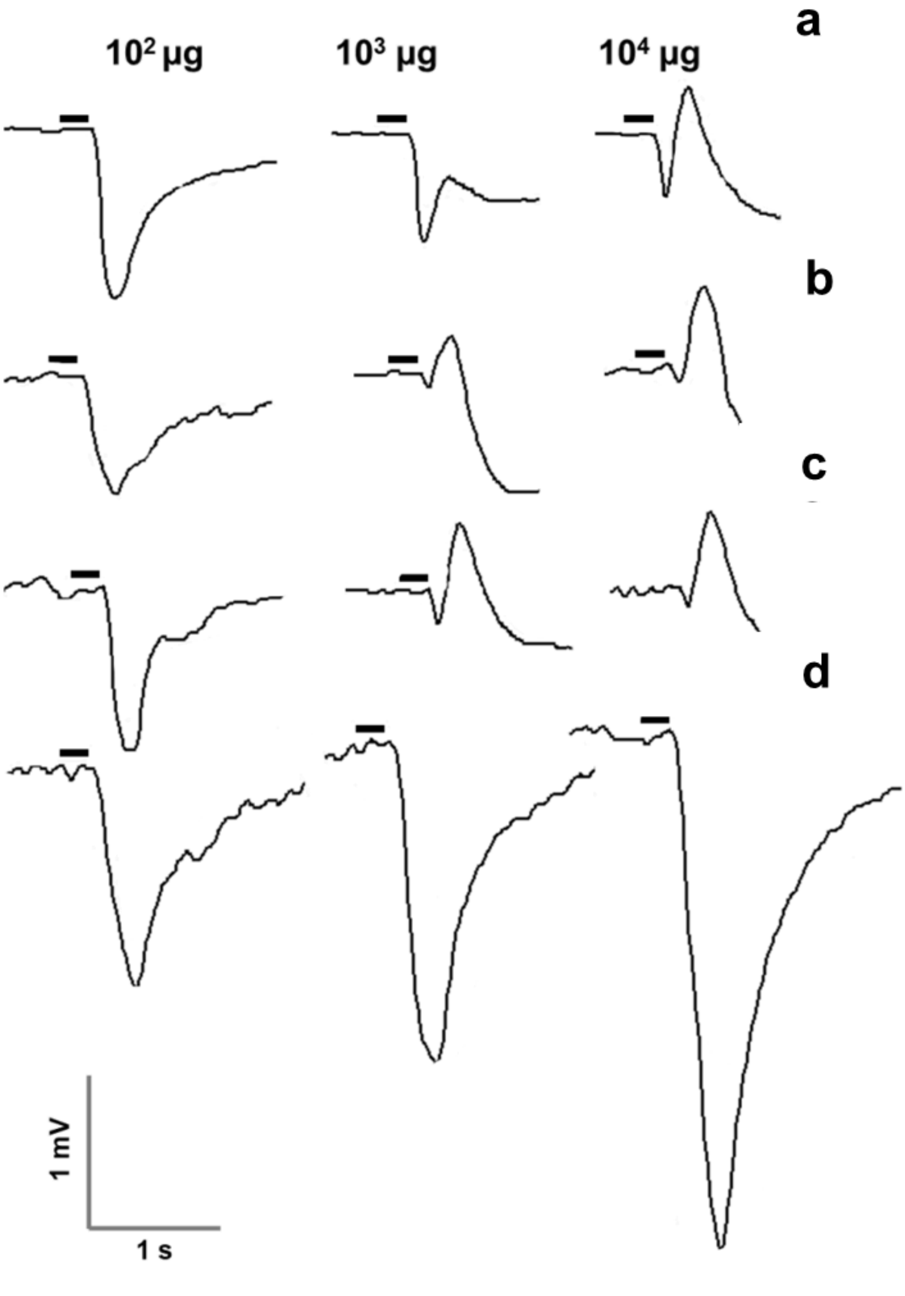

Figure S2. Representative EAG traces of *G. molesta* males in response to 10^2-10^4 µg of thyme oil (**a**), carvacrol (**b**), thymol (**c**), and *p*-cymene (**d**). Horizontal bars over traces indicate duration of the puffed stimulus.

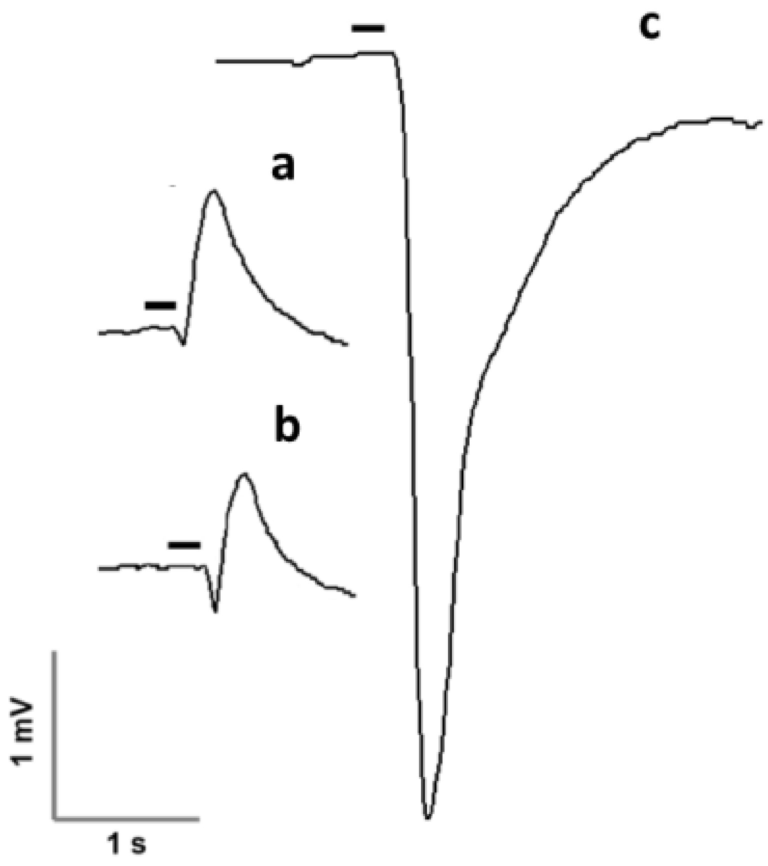

Figure S3. Comparative of EAG traces of *S. littoralis* males in response to thymol (10 mg) (**a**), carvacrol (10 mg) (**b**), and to a posterior stimulus of the major sex pheromone component *Z*9*E*11-14:OAc (1 µg) (**c**). Horizontal bars over traces indicate duration of the puffed stimulus. Similar V-shape profiles were recorded for *G. molesta* in response to sex pheromone.

Table S1. Sequential behavioral steps of *Grapholita molesta* males in wind tunnel when exposed to sex pheromone (PHE: *Z*8-12:OAc + *E*8-12:OAc + *Z*8-12:OH, 5 µg in 100:6:10 ratio), and when co-released with thyme oil (1:1 and 1:10), single components, and binary/ternary mixtures, according to their content in 50 µg of thyme oil (PHE, n = 60; other treatments, n= 30). Percentages followed by an asterisk are significantly different from that of the pheromone (Chi-square 2 x 2 test of independence with Yates´ correction, at α=0.05).

Test	Take-off	Halfway	Approach	Landing
**PHE**	98	94	93	93
**PHE + Thyme oil (1:1)**	100	100	100	83
**PHE + Thyme oil (1:10)**	97	97	97	69*
**PHE + *p*-cymene (15 µg)**	100	97	97	94
**PHE + carvacrol (1.5 µg)**	100	97	97	93
**PHE + thymol (22.5 µg)**	89	82	82	54*
**PHE + *p*-cymene (15 µg) + carvacrol (1.5 µg)**	100	100	100	86
**PHE + *p*-cymene (15 µg) + thymol (22.5 µg)**	100	100	100	84
**PHE + carvacrol (1.5 µg) + thymol (22.5 µg)**	100	100	97	76
**PHE + *p*-cymene (15 µg) + thymol (22.5 µg) + carvacrol (1.5 µg)**	100	84	84	75

Table S2. Sequential behavioral steps of *Spodoptera littoralis* males in wind tunnel when exposed to sex pheromone (PHE: *Z*9*E*11-14:OAc 10 µg), and when co-released with thyme oil (1:1 and 1:10), single components, and binary/ternary mixtures, according to their content in 100 µg of thyme oil (PHE, n = 56; other treatments, n= 30). Percentages followed by an asterisk are significantly different from that of the pheromone (Chi-square 2 x 2 test of independence with Yates´ correction, at α=0.05).

Test	Take-off	Halfway	Approach	Landing
**PHE**	100	93	88	84
**PHE + Thyme oil (1:1)**	100	90	70	60*
**PHE + Thyme oil (1:10)**	67*	63*	37*	23*
**PHE + *p*-cymene (30 µg)**	97	97	93	87
**PHE + carvacrol (3 µg)**	97	90	83	73
**PHE + thymol (45 µg)**	93	83	67*	43*
**PHE + *p*-cymene (30 µg) + carvacrol (3 µg)**	100	93	87	77
**PHE + *p*-cymene (30 µg) + thymol (45 µg)**	97	97	87	80
**PHE + carvacrol (3 µg) + thymol (45 µg)**	93	90	73	50*
**PHE + *p*-cymene (30 µg) + thymol (45 µg) + carvacrol (3 µg)**	97	97	87	60*
